# Supplementary material for: Deletion of wfs1 Impairs Oligodendrocyte Precursor Cells Dorsal Distribution and Myelination Through the wfs1-hmgcs1 Axis in Zebrafish
Source: Biology (Basel). 2026 Aug 16;15(16):1402. doi: 10.3390/biology15161402 (PMC13510151; doi:10.3390/biology15161402)
Supplement: Supplementary file 1 [file biology-15-01402-s001.zip › File S1-Transcriptome sequencing data and differentially expressed genes (DEGs) in wfs1-- zebrafish at 5 dpf/2.KEGG/wfs1KOvsWT/DOWN/src/dre04910.html]

dre04910


- dre:559001

- Down regulated genes

559001\_1(-1.11683329469951)

- dre:100001260

- Down regulated genes

100001260(-10.2224161507053)

- dre:79380

- Down regulated genes

79380\_1(-1.05168581152728)

- dre:58100

- Down regulated genes

58100\_1(-4.1286149197908)

- dre:100000252

- Down regulated genes

100000252\_1(-1.7718817168434)

- dre:406339

- Down regulated genes

406339\_1(-1.38680349052238)

Close
